# Twinning-mediated formability in Mg alloys

**DOI:** 10.1038/srep22364

**Published:** 2016-03-01

**Authors:** Byeong-Chan Suh, Jae H. Kim, Ji Hyun Hwang, Myeong-Shik Shim, Nack J. Kim

**Affiliations:** 1 Graduate Institute of Ferrous Technology and Centre for Advanced Aerospace Materials, Pohang University of Science and Technology, Pohang 790-784, Korea

## Abstract

Mg alloys are promising candidates for automotive applications due to their low density and high specific strength. However, their widespread applications have not been realized mainly because of poor formability at room temperature, arising from limited number of active deformation systems and strong basal texture. It has been recently shown that Mg-Zn-Ca alloys have excellent stretch formability, which has been ascribed to their weak basal texture. However, the distribution of basal poles is orthotropic, which might result in anisotropy during deformation and have adverse effect on formability. Here, we show that tension twinning is mainly responsible for enhanced formability of Mg-Zn-Ca alloys. We found that tension twinning is quite active during both uniaxial deformation and biaxial deformation of Mg-Zn-Ca alloy even under the stress conditions unfavourable for the formation of tensile twins. Our results provide new insights into the development of Mg alloys having high formability.

In recent years, the demand for lightweighting of automobiles has been increased to tackle the global issues of energy conservation and environmental protection^1^,^2^,^3^. Being the lightest of structural alloys, Mg alloys offer significant potential for weight reduction, but have yet to see significant application in automobiles, particularly in sheet form^2^,^3^. One of the major drawbacks preventing such application is their poor formability at room temperature, originating from their strong basal texture and limited number of slip systems^3^,^4^. Therefore, numerous studies have been conducted to improve the formability of Mg alloys by weakening/randomizing the texture and activating non-basal deformation modes^5^,^6^,^7^,^8^,^9^,^10^,^11^,^12^,^13^,^14^,^15^,^16^,^17^,^18^,^19^. One of the most important findings from such studies is that the rare earth elements (REEs) containing Mg alloys generally show better formability than other non-REEs containing Mg alloys. It has been suggested that good formability of the REEs containing Mg alloys comes from their weak/random texture^5^,^6^,^8^,^10^,^20^,^21^ and the additional activation of non-basal slip modes such as prismatic and pyramidal slip besides basal slip^21^,^22^,^23^. However, the high cost and scarcity of REEs would pose a problem for application of REEs containing Mg alloys as structural components of automobiles.

It has been recently reported that the addition of Ca to Mg-Zn alloys also results in a weak/random texture and high stretch formability^11^,^12^,^13^,^14^,^15^,^24^. Interestingly, the textures of these REEs and Ca containing alloys are quite similar, which can be characterized by the broadened angular distribution and split of basal poles along the transverse direction (TD) as well as weak basal pole intensity. Although the weak/random texture can give high formability in these alloys, their texture is in fact less than ideal such that their basal poles are oriented mostly along one direction having an orthotropic texture. In such a case, one can expect that there would be severe in-plane anisotropy during uniaxial tensile deformation along different directions and biaxial deformation experienced during stretch forming. In the case of REEs containing alloys, additional operation of non-basal slip such as prismatic and pyramidal slip^21^,^22^,^23^ can certainly play as an additional factor responsible for their high stretch formability. On the other hand, although the operation of prismatic and pyramidal slip has not been reported for Mg-Zn-Ca alloys, Mg-Zn-Ca alloys are known to have high tension twinning activity^25^, which might be responsible for their high stretch formability. However, considering that tension twinning can occur only under certain conditions, i.e., operation of tensile stress parallel to c-axis and compressive stress perpendicular to c-axis of Mg crystal, it is not clear whether tension twinning can occur homogeneously during stretch forming of Mg-Zn-Ca alloys having orthotropic texture and enhance their stretch formability.

In the present work, we investigated the tension twinning behaviour of Mg-1Zn-1Ca alloy during uniaxial tensile test and stretch forming to understand the origin of high stretch formability of Mg-Zn-Ca alloys.

## Results

### Microstructure and texture before deformation

**Figure 1:**
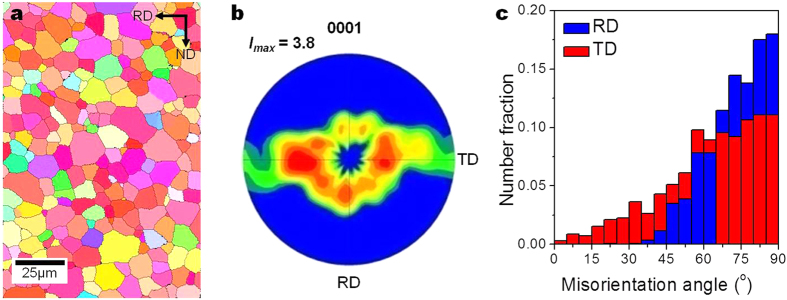
Microstructure and texture of ZX11 alloy. (**a**) EBSD inverse pole figure (IPF) map, (**b**) (0001) pole figure, and (**c**) misorientation angles between the c-axes of grains and the RD and TD. The average grain size of the alloy is ~10.8 μm and the micro-texture of the alloy shows the broadened angular distribution of basal poles as well as a splitting of basal poles tilted 30° ~ 60° from the ND towards the TD. There is also a splitting of basal poles tilted 35° ~ 40° from the ND towards the RD. Only a very small fraction (less than 2%) of the grains have their c-axes within ±45° from the RD, while the fraction of the grains whose c-axes are within ±45° from the TD is about 18%.

The microstructure and micro-texture of ZX11 alloy before deformation are shown in Fig. 1a,b, respectively. The average grain size of the alloy is ~10.8 μm. The micro-texture of the alloy is quite similar to the texture measured by X-ray diffraction^11^, which can be described as the broadened angular distribution of basal poles from the normal direction (ND) towards the TD as well as a splitting of basal poles tilted 30° ~ 60° from the ND towards the TD. There is also a splitting of basal poles tilted 35° ~ 40° from the ND towards the rolling direction (RD). The misorientation angles between the c-axes of grains and the RD and ND were measured as shown in Fig. 1c. It shows that there is only a very small fraction (less than 2%) of the grains whose c-axes are within ±45° from the RD. On the other hand, the fraction of the grains whose c-axes are within ±45° from the TD is about 18%.

### Mechanical properties and anisotropy

**Figure 2:**
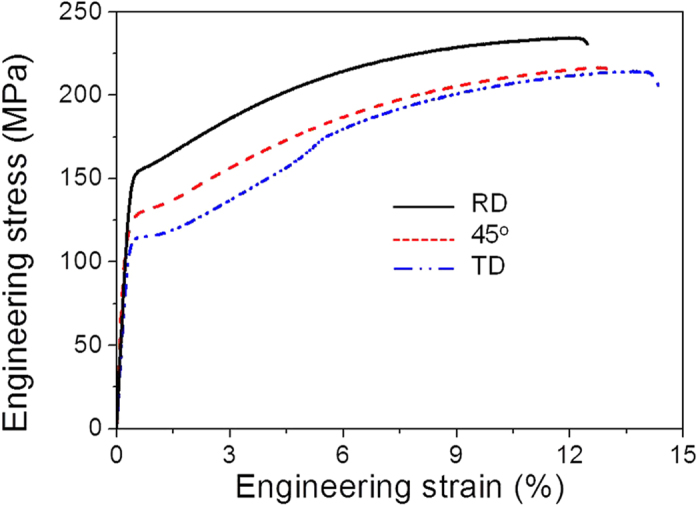
Engineering stress-strain curves of the alloy along the RD, 45° to the RD, and the TD. Both yield and tensile strengths are highest along the RD, followed by 45° to the RD, and the TD, while elongation values show the opposite trend. The stress-strain curves have sigmoidal shape, mostly evident along the TD, suggesting that tension twinning is quite active at an early stage of tensile deformation along the TD.

Engineering tensile stress-strain curves of the alloy subjected to tensile tests along various directions are shown in Fig. 2. The yield strength, tensile strength, and elongation were measured from the curves, and the results are summarized in Table 1. As expected from the alloy’s orthotropic texture, the alloy shows quite different tensile properties depending on tensile loading direction as shown in Fig. 2 and Table 1. It shows that both yield and tensile strengths are highest along the RD, followed by 45° to the RD, and the TD, while elongation values show the opposite trend. It also shows that the stress-strain curves have sigmoidal shape, mostly evident along the TD, suggesting that tension twinning is quite active at an early stage of tensile deformation along the TD^26^.

It is now established that the stretch formability of Mg alloy sheets is dependent on their ability to deform along the thickness direction^10^, which can be described by the Lankford value (*r*-value), *r* = *ε*_*w*_/*ε*_*t*_. The *r*-values have been measured along three different directions and the results are summarized in Table 1. It shows that the average *r*-value of the present alloy is 1.21, which is much smaller than that (~3.0) of AZ31 alloy^27^. Although the alloy shows small average *r*-value, however, there is a difference in *r*-values depending on tensile loading directions as expected from the orthotropic texture, with planar anisotropy of *r*-value (Δ*r*) of 0.42, which is somewhat larger than those of other Mg alloys^10^,^28^,^29^. Despite the differences in *r*-values and strengths depending on tensile loading directions, ZX11 alloy shows the Index Erichsen (IE) value of 8.8 mm, which is much larger than that of AZ31 alloy at the same yield strength level^5^. It is also interesting to note that ZX11 alloy shows high IE value, despite the presence of rather coarse (~1 μm) precipitates in the microstructure^11^, suggesting that the precipitates have a minor effect on the stretch formability, similar to the results of Yuasa *et al.*^30^. The thickness strain at the top of the dome after stretch forming was measured at dome heights of 4.2 mm and 8.8 mm (after failure). The thickness strain of ZX11 alloy at a dome height of 4.2 mm is 5.7%, which is slightly larger than that (3%) reported for AZ31 alloy at a dome height of 3.2 mm^31^. However, the thickness strain of ZX11 alloy becomes 27.1% after failure, indicating an occurrence of significant deformation along the thickness direction during stretch forming of ZX11 alloy.

### Twinning behaviour under uniaxial tensile deformation

**Figure 3:**
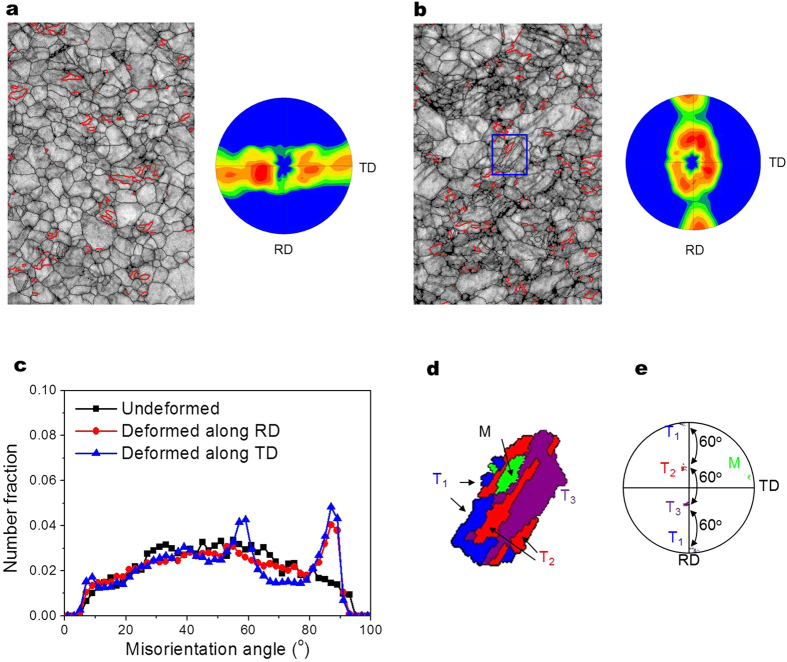
Microstructural changes during tensile deformation. (**a**) IQ map of the RD-loaded ZX11 alloy and its (0001) pole figure, (**b**) IQ map of the TD-loaded ZX11 and its (0001) pole figure, (**c**) Misorientation profiles of ZX11 alloy before and after deformation, (**d**) enlarged IPF map of the boxed area in (**b**), and (**e**) (0001) pole figure of twins and matrix shown in (**d**). Note that different twin variants are marked with different colours.

Microstructure of the alloy after tensile deformation (10% strain) has been investigated to understand its twinning behaviour. Figure 3a,b show electron backscatter diffraction (EBSD) image quality (IQ) maps after tensile deformation along the RD and TD, respectively. It can be seen that there are formations of tension twins (whose boundaries are marked by red lines) in both cases. It also shows that the texture changes after tensile deformation; the angular distribution of basal poles becomes broadened along the TD in the RD-loaded tensile specimen and along the RD in the TD-loaded tensile specimen as shown in Fig. 3a,b (insets), respectively. The distribution of misorientation angles after tensile deformation has been measured as shown in Fig. 3c. After tensile deformation, both the RD-loaded and TD-loaded tensile specimens show a large increase in the number fraction at the misorientation angle of about 86°, indicating an active formation of tension twins during tensile deformation along both the RD and TD. It is interesting to notice that there is an active tension twinning in the RD-loaded tensile specimen, despite the unfavourable orientations of most grains for tension twinning when loaded along the RD, i.e., the misorientation angles between the c-axes of most grains and the RD are larger than 45° (Fig. 1c). While the number fractions at the misorientation angle of about 86° are similar between the RD-loaded and TD-loaded specimens, interestingly, the TD-loaded specimen additionally shows a relatively large number fraction at the misorientation angle of 60° (Fig. 3c). Such misorientation angle is possible when there is a formation of several variants of tension twins within one grain^32^,^33^ as shown in Fig. 3d, the enlarged inverse pole figure (IPF) map of the area marked by blue rectangle in Fig. 3b. It shows that this particular grain contains three variants (marked T_1_, T_2_, and T_3_) of tension twins, which are all rotated 86° along <11–20> from the matrix (marked M) whose c-axis is nearly parallel to the TD. These twins are also rotated 60° along <1–100> with respect to the neighbouring twins as shown in Fig. 3e. When the grain with its c-axis nearly parallel to the TD is subjected to tensile loading along the TD, the Schmid factors (SFs) of these three tension twin variants are almost same and quite high, and therefore these three tension twin variants can be generated having an angular relationship of 60° with each other.

### Twinning behaviour under biaxial deformation

**Figure 4:**
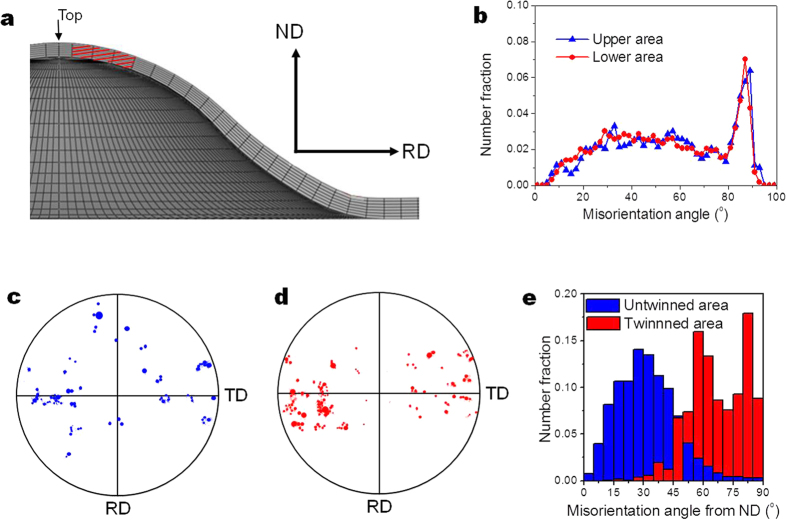
Microstructural changes during stretch forming. (**a**) Schematic drawing showing the RD-ND cross section of the stretch formed specimen (the observed area is red-shaded), (**b**) misorientation profiles after stretch forming of the red-shaded area in (**a**), (**c**) (0001) pole figure of tension twins formed in the upper area, (**d**) (0001) pole figure of tension twins formed in the lower area, and (**e**) misorientation angles between the c-axes of the twinned and untwinned areas located at near-top area (red-shaded area in (**a**) and the ND.

To examine the twinning behaviour during stretch forming, the RD-ND cross section at near-top area (red-shaded area in Fig. 4a) of the stretch formed specimens have been analysed by EBSD. Since the stress state can be quite different through the thickness of sheet during stretch forming and therefore twinning behaviour, the cross section was divided into two areas; upper area and lower area. Figure 4b shows the distribution of misorientation angles in the upper and lower areas after deformation to a dome height of 4.2 mm, as shown as triangle and circle markers, respectively. After stretch forming, the upper area shows a large fraction of misorientation angles near 86° (marked by triangles in Fig. 4b). The lower area also shows a large fraction of misorientation angles near 86° (marked by circles in Fig. 4b), which is similar to that observed in the upper area. These results indicate that there is a formation of a large amount of tension twins after deformation to a dome height of 4.2 mm, regardless of the area through the thickness of the specimen.

However, there is a difference in the orientation of tension twins formed in the upper and lower areas. As shown in Fig. 4c,d, the c-axes of tension twins formed in the upper area are spread from the ND towards the both RD and TD, while those in the lower area are spread from the ND mostly towards the TD. These results suggest that tension twins formed in the upper area are due to the operation of different stress states from that in the lower area during stretch forming. The misorientation angles between the c-axes of the grains located at near-top area (red-shaded area in Fig. 4a) and the ND were calculated as shown in Fig. 4e. It shows that the c-axes of tension twins (i.e., twinned area) are oriented away from the ND more than those of untwinned area.

## Discussion

The above mentioned results show that there is a significant activity of tension twinning in ZX11 alloy during both uniaxial tensile deformation (regardless of tensile loading directions) and biaxial stretch forming (Figs 3c and 4b).

It is well known that tension twinning can occur when tensile stress is applied parallel to c-axis or compressive stress perpendicular to c-axis of Mg grain^34^,^35^. Since there is a large number of grains whose c-axes are oriented towards the TD (i.e., smaller than 45°) as shown in Fig. 1b,c, active tension twinning can occur when the specimen is subjected to tensile deformation along the TD. On the other hand, the formation of tension twins upon tensile deformation along the RD, which places the c-axes of most grains in unfavourable orientations for tension twinning (i.e., larger than 45°) as shown in Fig. 1b,c, can occur as a result of the operation of compressive stress along the width direction (i.e., TD) due to constraint by neighbouring grains, as observed in the similarly textured Mg-4Zn-1Gd alloy^36^. The area fraction of tension twins in the unfavourably oriented RD-loaded specimen after 10% strain is only a bit smaller than that in the TD-loaded specimen (6.9% vs. 8.3%), but much larger than those observed in AZ31 alloy^31^,^37^ and Mg-4Zn-1Gd alloy^36^, suggesting that the addition of Ca promotes the formation of tension twins. This is in agreement with the results of Nakano *et al.* showing that tension twinnability is enhanced by the addition of Ca because of the increased unstable stacking fault energy and decreased unstable twin fault energy^25^.

**Figure 5:**
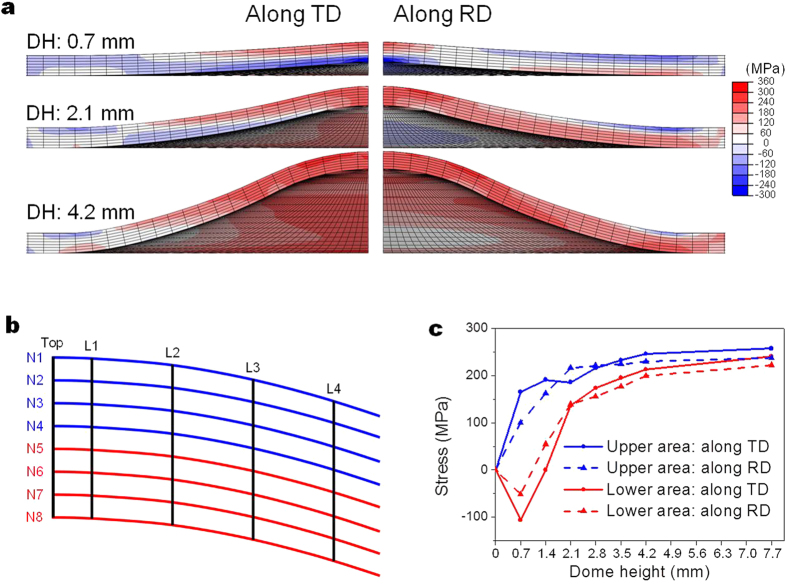
Finite element (FE) simulation for stretch forming. (**a**) Variation in stress distribution along the RD and TD with dome height (red and blue colours indicate tensile and compressive stresses, respectively), (**b**) schematic drawing showing the RD-ND cross section of the stretch formed specimen subjected to FE simulation, and (**c**) variation in stress with dome height. DH: dome height.

However, unlike the case of uniaxial tensile test, the stress state during stretch forming is mostly biaxial and might not be favourable for the formation of tension twins uniformly across the sheet plane of ZX11 alloy having orthotropic texture. Nevertheless, the analysis of stretch formed sheet shows that tension twinning is quite active through the thickness of the sheet during stretch forming (Fig. 4b). Interestingly, the orientation of tension twins formed in the upper area has been found to be different from that in the lower area as shown in Fig. 4c,d. Finite element simulation was conducted to understand the stress evolution during stretch forming and its effect on the tension twinning behaviour. Figure 5a shows the variation in stress distributions along the RD and TD with the dome height of ZX11 alloy. It shows that at an early stage of stretch forming (as shown at the dome height of 0.7 mm), compressive stress develops along the both RD and TD in the lower area of the dome, but is more extended across the lower area along the TD than along the RD. Compressive stress developed in the lower area is eventually replaced by tensile stress with increasing deformation (as shown at the dome height of 2.1 mm). In the upper area, on the other hand, tensile stress develops along the both RD and TD since the start of stretch forming. Similar to the case of the lower area, tensile stress is more extended across the upper area along the TD than along the RD. To have a quantitative information on stress distribution, stress level at near-top area (red-shaded area in Fig. 4a and L1-L4 in Fig. 5b) of the dome was measured. Figure 5c shows the variation in average stresses in the upper and lower areas with dome height. Here, the upper area was defined as N1-N4 and the lower area N5-N8 in Fig. 5b. It shows that in the lower area, compressive stress operates up to a larger dome height along the TD than along the RD and the magnitude of compressive stress is higher along the TD than along the RD. Compressive stress is eventually replaced by tensile stress as the dome height increases, and during a later stage of stretch forming the magnitude of tensile stress becomes higher along the TD than along the RD. In the case of the upper area, the magnitude of tensile stress is higher along the TD than along the RD, except at the dome heights between ~1.4 mm and ~2.8 mm.

**Figure 6:**
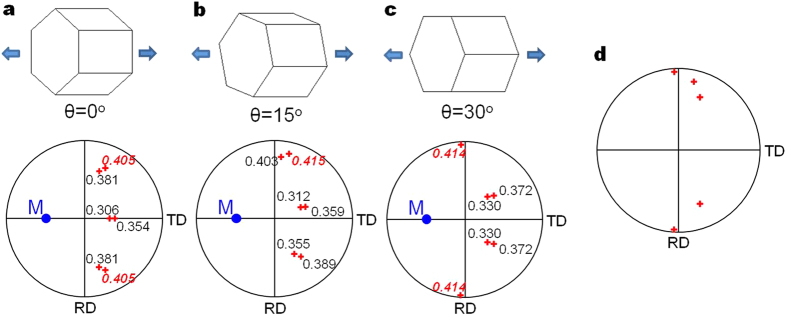
Schmid factor analysis on tension twin variants formed from the grain with *c*-axis located at M (50° tilted towards the TD) under tensile stress along the TD. Since the *a*-axis can be randomly oriented, the angle *θ* between *a*-axis and loading axis can vary in the range of 0–60°; however, only the angle of *θ* in the range of 0–30° can be considered because of the unique angle relationship between three *a*-axes of hcp Mg lattice^33^. (**a**) *θ* = 0°, (**b**) *θ* = 15°, (**c**) *θ* = 30°, and (**d**) possible locations of c-axes of tension twins. In (**a–c**), the locations of possible tension twin variants (i.e., having the highest SFs for each case) are shown in bold and italic typeface with red colour.

The above analysis shows that at an early stage of stretch forming, the most dominant stress in the lower area is compressive stress along the TD and the one in the upper area is tensile stress along the TD. As shown in Fig. 1b,c, the initial texture of the alloy shows the high intensity of basal poles within ±45° from the ND towards the TD, indicating the presence of many grains that can form tension twins upon the operation of compressive stress along the TD. It is well known that for compression perpendicular to the c-axis of the parent grain, the c-axis of the formed tension twin is oriented within ~±30° towards the direction of compressive stress^33^ and therefore the c-axes of the tension twins formed at the lower area would be oriented towards the TD as shown in Fig. 4d. Although the operation of compressive stress along the RD would produce tension twins with their c-axes oriented towards the RD, it is quite unlikely to occur since compressive stress along the TD is about 2 times higher than along the RD. In the case of the upper area, tensile stress along the RD is not expected to form tension twins since the misorientation angles between the c-axes of almost all grains and the RD are larger than 45°. This is different from twinning behaviour observed in uniaxially deformed specimen (Fig. 3a) since biaxial tensile stress operates during stretch forming. However, tension twins can form under tensile stress along the TD since the basal poles of the alloys are broadened along the TD. In such a case, the c-axes of the formed tension twins would be aligned perpendicular to the TD as shown in Fig. 4c since for tension nearly parallel to the c-axis of the parent grain, the c-axis of the formed tension twin is oriented perpendicular to the c-axes of parent grain^33^, as schematically shown in Fig. 6.

The above analyses show that tension twinning is quite active even during stretch forming of the asymmetrically textured ZX11 alloy, and can improve its stretch formability. It has been reported that grain coarsening can improve the stretch formability of Mg alloy^5^,^31^. Chino *et al.* studied the effect of grain size (5.6 μm, 10.4 μm, 17.0 μm and 31.0 μm) on the stretch formability of AZ31 alloy and showed that such behavior is due to enhanced twinning activity associated with grain coarsening, which is more evident when the grain size becomes larger than 30 μm^31^. However, the present ZX11 alloy has a fine grain size of 10.8 μm and therefore its high twinning activity and high stretch formability are not attributed to its grain size.

As shown in Fig. 4c,d, the orientations of tension twins are more random than those of parent grains, with their c-axes spreading from the ND towards the RD and TD. As shown in Fig. 4e, the c-axes of almost all tension twins (i.e., twinned area) are oriented more than 45° from the ND (i.e., thickness direction), while those of the untwinned area are within 45° from the ND, indicating that the basal planes of tension twins are favourably oriented for the deformation along the thickness direction. Since the stress level is higher along the TD than along the RD during a later stage of stretch forming, the SFs for basal slip were calculated along the TD. It shows that the average SF for basal slip of tension twins is higher than that of the untwinned area (0.35 *vs* 0.28). It is worth noting that the thickness strain at the top of the dome increases from 5.7% to 27.1% at dome heights of 4.2 mm and 8.8 mm (after failure), respectively, indicating that an increase in the thickness strain at a later stage of stretch forming is related to the deformation in tension twins. The inclination of basal planes towards the thickness direction and high SFs for basal slip of tension twins can induce deformation along the thickness direction, improving stretch formability of ZX11 alloy.

In short, an enhanced tension twinning activity in ZX11 alloy modifies its microstructure such that the grains which previously could not deform along the thickness direction can deform along the thickness direction after twinning, which is vital for high stretch formability in Mg alloys. These findings provide a new alloy design route to wrought Mg alloys, demonstrating that high stretch formability can be obtained in Mg alloys by promoting the activity of tension twinning. Furthermore, such enhanced twinning activity is obtainable by the use of an alloying element, Ca, other than REEs.

## Methods

An alloy with a nominal composition of Mg-1Zn-1Ca (wt%), ZX11, was fabricated by a twin-roll casting process. Details of the TRC process are described elsewhere^11^,^38^,^39^. After homogenization at 713 K for 1 h followed by water quenching, the alloy was hot rolled at 573 K with a total reduction of 50% (5 passes; 13% reduction per pass). After rolling, the alloy was given final annealing at 713 K for 30 min followed by water quenching. Microstructure was observed by electron backscatter diffraction (EBSD). (0001) pole figures were calculated by the discrete binning method with a bin size of 5° from EBSD data. Tensile tests were conducted using the specimens with a gauge length of 12.5 mm, a gauge width of 5 mm, and a gauge thickness of 1 mm at a strain rate of 6.4×10^−4^ s^−1^. The Lankford value, *r*-value (*ε*_*w*_*/ε*_*t*_), has also been measured after 10% tensile strain. Stretch formability was evaluated by the Erichsen test (disc shape specimen with 50 mm diameter). The punch diameter and speed used were 27.5 mm and 0.5 mm s^−1^, respectively. Silicon oil was used as a lubricant.

In addition, a three-dimensional finite element (FE) simulation was conducted under ABAQUS 6.10/Explicit code to analyse the stress distribution during stretch forming. The FE model consisted of four components: a punch, a lower die, a holder, and a blank sheet. The tool components, i.e., punch, die and holder, were considered to be analytical rigid bodies. Only one quarter of the blank sheet was modeled considering symmetrical boundary conditions of Erichsen test. The blank was meshed with 4-node reduced integration shell element, 4SR. There were 5 integration points through thickness direction in an element. Mesh size of 0.8 mm along radial direction was adopted. The lower die was fixed in all directions and the punch was moved following the prescribed displacement condition. A constant holding force of 10 kN to the holder was applied. Coulomb’s constant friction law was assumed and the friction constant between the tool components and blank was assumed as 0.1. The elastic modulus and Poisson’s ratio used were 45 GPa and 0.25, respectively. For simplicity, isotropic hardening law was assumed and Voce hardening law was adopted. To take into account the anisotropy of ZX11 alloy, non-quadratic anisotropic yield function, Yld2000-2d, was used.

## Additional Information

**How to cite this article**: Suh, B.-C. *et al.* Twinning-mediated formability in Mg alloys. *Sci. Rep.* **6**, 22364; doi: 10.1038/srep22364 (2016).

## Figures and Tables

**Table 1:** Summary of the tensile test data.

Tensile direction	YS (MPa)	UTS (MPa)	Elongation (%)	*r*-value	*r*_avg_	Δ*r*	IE (mm)
RD	154.9	230.6	12.3	1.21			
45^°^	128.4	216.5	12.5	1.49	1.21	0.42	8.8
TD	114.0	214.4	13.6	0.93			

YS: yield strength, UTS: ultimate tensile strength, *r*_avg_: average *r* value, Δ*r*: planar anisotropy, and IE: Erichsen value.
